# Temperature-Dependent Changes in Resolution and Coercivity of Superparamagnetic and Superferromagnetic Iron Oxide Nanoparticles

**DOI:** 10.18416/IJMPI.2023.2303056

**Published:** 2023-03-19

**Authors:** Owen Doyle, Jacob Bryan, Melissa Kim, Chinmoy Saayujya, Sophie Nazarian, Javier Mokkarala-Lopez, Renesmee Kuo, Mariam Yousuf, Prashant Chandrasekharan, Benjamin Fellows, Steven Conolly

**Affiliations:** a Department of Bioengineering, UC Berkeley, Berkeley CA, USA; b Department of Electrical Engineering and Computer Sciences, UC Berkeley, Berkeley CA, USA; c Magnetic Insight, Alameda CA, USA

## Abstract

Magnetic Particle Imaging (MPI) is a tracer-based imaging modality with immense promise as a radiation-free alternative to nuclear medicine imaging techniques. Nuclear medicine requires “hot chemistry” wherein radioactive tracers must be synthesized on-site, requiring expensive infrastructure and labor costs. MPI’s magnetic nanoparticles, superparamagnetic iron oxide nanoparticles (SPIOs), have no significant signal decay over time which removes cost barriers associated with nuclear medicine studies such as FDG-PET. While SPIOs are the current industry standard MPI tracer, recent developments in synthesizing superferromagnetic iron oxide nanoparticles (SFMIOs) and high resolution SPIOs (HR-SPIOs), a new class of nanoparticle with almost zero coercivity, have yielded a 30-fold improvement in resolution (0.4 mT) and SNR. To better understand the long-term performance of these new nanoparticles, this investigation reports changes in SPIO (VivoTrax Plus), HR-SPIO, and SFMIO resolution, along with SFMIO coercivity, at low temperatures (−2, 2 °C) and room temperature (18–22 °C) over 12 weeks. We find that changes in HR-SPIO resolution are more sensitive to storage temperature than SFMIOs. Additionally, we observe no appreciable difference in SFMIO coercivity between the two temperatures over time. These results can inform research on optimizing tracer synthesis while lending practical information to future hospitals about the highly accessible conditions for the transit and storage of tracers.

## I. Introduction

**Figure 1:**
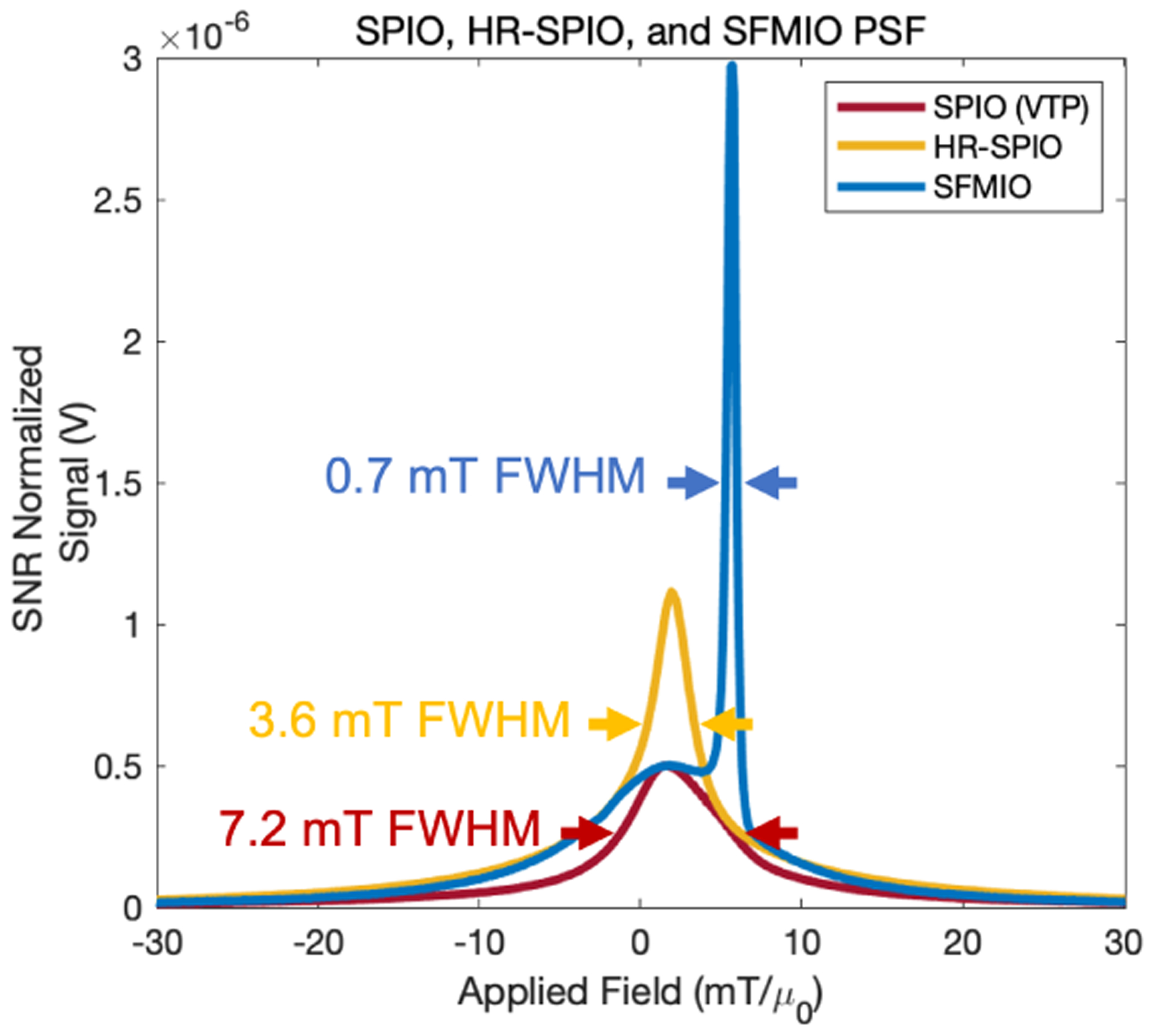
Point spread functions (PSFs) of a commercial SPIO (VivoTrax Plus), an HR-SPIO, and an SFMIO tracer. PSFs are slew-rate normalized and generated by a 10 mT, 20 kHz transmit field. HR-SPIOs have improved resolution compared to SPIOs. Note the characteristic shift of the SFMIO peak from the FFP center. The applied field at the SFMIO peak signal is recorded as the coercivity.

**Figure 2:**
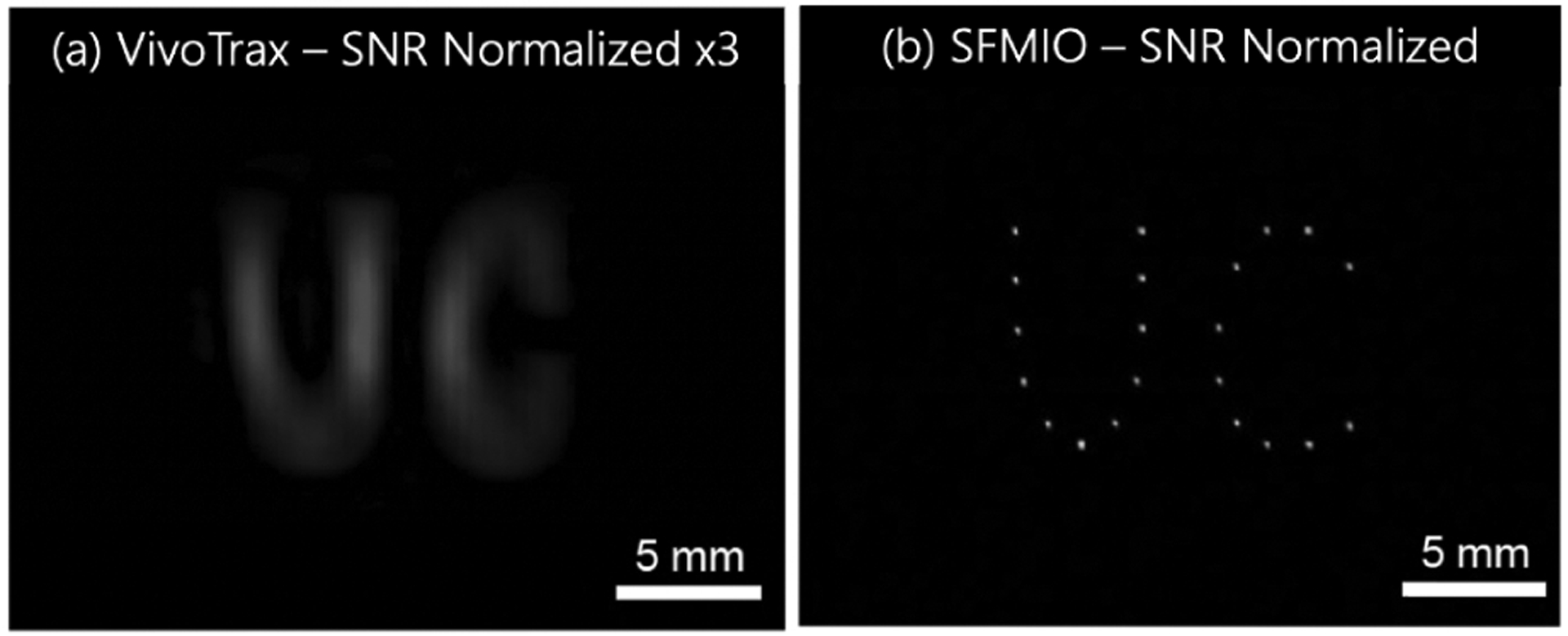
SFMIOs show a 30-fold boost in resolution and SNR experimentally. (a) MPI images of pointillism UC-phantom made out of a standard tracer (VivoTrax) filled in capillary tubes, shown at 3-times actual brightness. (b) The same phantom was scanned using lab-synthesized SFMIOs with 0.8 mT resolution. Notice that the points are blurred and unresolved for the Vivo-Trax tracers.

Magnetic particle imaging (MPI) utilizes magnetic nanoparticle tracers that can be safely administered inside the body to perform high-contrast physiological imaging [1, 2]. Once inside the magnetic field generated by an MPI scanner, the tracer magnetization elicits a detectable signal used to determine their location within a gradient. The tracer magnetization response and the gradient field strength determine the image resolution [3]. Superparamagnetic iron oxide nanoparticles (SPIOs) have reached resolution limits of around 1 mm with current scanner hardware [4]. However, recent developments in Superferromagnetic iron oxide nanoparticles (SFMIOs) have enabled an order of magnitude improvement in image resolution due to their step-like magnetization response [5]. This boost in SFMIO resolution is due to hysteresis arising from magnetic dipole interactions between individual iron oxide nanoparticles. These interactions result in mesoscale chain formation, a phenomenon not observed in SPIOs. Magnetization reversal in SFMIO chains occurs at a coercive threshold, which shifts their peak signal to a non-zero applied field. On the other hand, high resolution SPIOs (HR-SPIOs) exhibit a similar resolution boost but with negligible coercivity (Figure 1). These developments have produced 30-fold improvements in image resolution and SNR (Figure 2), drastically advancing the potential of clinical MPI.

Through controlled synthesis and oxidation techniques, the process of creating HR-SPIOs and SFMIOs has become much more predictable [6]. Controllable oxidation of the magnetic “dead layer” produces nanoparticles that are richer in magnetite (Fe_3_O_4_), which is highly magnetized [7]. While great strides in tracer synthesis have been made, there is less inquiry about the conditions that influence the longevity of high resolution nanoparticles. To begin facing this question, our study evaluates the effect of storing HR-SPIOs and SFMIOs at different temperatures.

## II. Methods and Materials

Six laboratory-synthesized nanoparticles (3 HR-SPIOs and 3 SFMIO), in addition to VivoTrax Plus (VTP; Magnetic Insight, USA), were analyzed over 12 weeks. Particles were synthesized via thermal decomposition using an extended lamer mechanism [8] with a post-synthesis oxidation step [6]. The particles were synthesized at different times ranging from zero to nine weeks prior to the start of the experiment and stored at −2 °C. All lab-synthesized particles have an oleic acid surface coating and are suspended in toluene [9]. VTP, which has carboxydextran-coated particles suspended in a phosphate-buffered saline (PBS) buffer, was stored within the recommended temperature range at 2 °C. VTP is kept at a higher temperature due to the higher freezing point of PBS compared to toluene.

Measurements were made using an arbitrary waveform relaxometer (AWR), an instrument designed for high throughput nanoparticle analysis [10]. Nanoparticles were excited at a transmit frequency of 20 kHz and transmit field amplitudes between 2–40 mT were tested in each recording session to achieve the lowest full width at the half-maximal value (FWHM) of the point spread function (PSF). PSFs were constructed using the MPI X-Space reconstruction algorithm [11]. While SPIO PSFs peak at the field free point (FFP), when the gradient field is 0 mT, the peak of SFMIO PSFs exhibit a characteristic shift from the FFP which corresponds to their magnetic coercivity (Figure 1). In this study, we report the lowest FWHM as the particle resolution and the applied field magnitude at the SFMIO PSF peaks as the coercivity. All PSFs were analyzed after normalizing for the slew rate of the transmit field. The coercivity is recorded for the PSF with the lowest FWHM among a scan of multiple transmit amplitudes. Periodic measurements were made to track the progression of each particle’s resolution and, for SMFIOs, coercivity.MPI images of point phantoms (capillary tube filled with tracers) were obtained in our field-free line 6.3 T/m gradient MPI scanner using a 20 mT/20 kHz excitation field (Figure 2). Images were reconstructed using the x-space algorithm and represented after normalization [5, 12–14].

## III. Experiments

To investigate the effect of temperature on nanoparticle degradation, each particle had a sample stored at a low temperature and at room temperature. In the first set, lab-synthesized particles were stored in the freezer at −2 °C while VTP was stored in the fridge at 2 °C. In the other set, all particles were stored together at approximately 18–22 °C. VTP nanoparticles were suspended in PBS buffer while lab-synthesized particles were suspended in toluene.

Samples of 40 μL were stored in glass PCR tubes with crimped caps. Without the addition of inert gas, the oxidative conditions are equal among all samples. The same samples were used throughout the study without replacement. Additionally, Parafilm was wrapped around the cap for a tighter seal and better fit in the AWR bore.

## IV. Results

**Figure 3:**
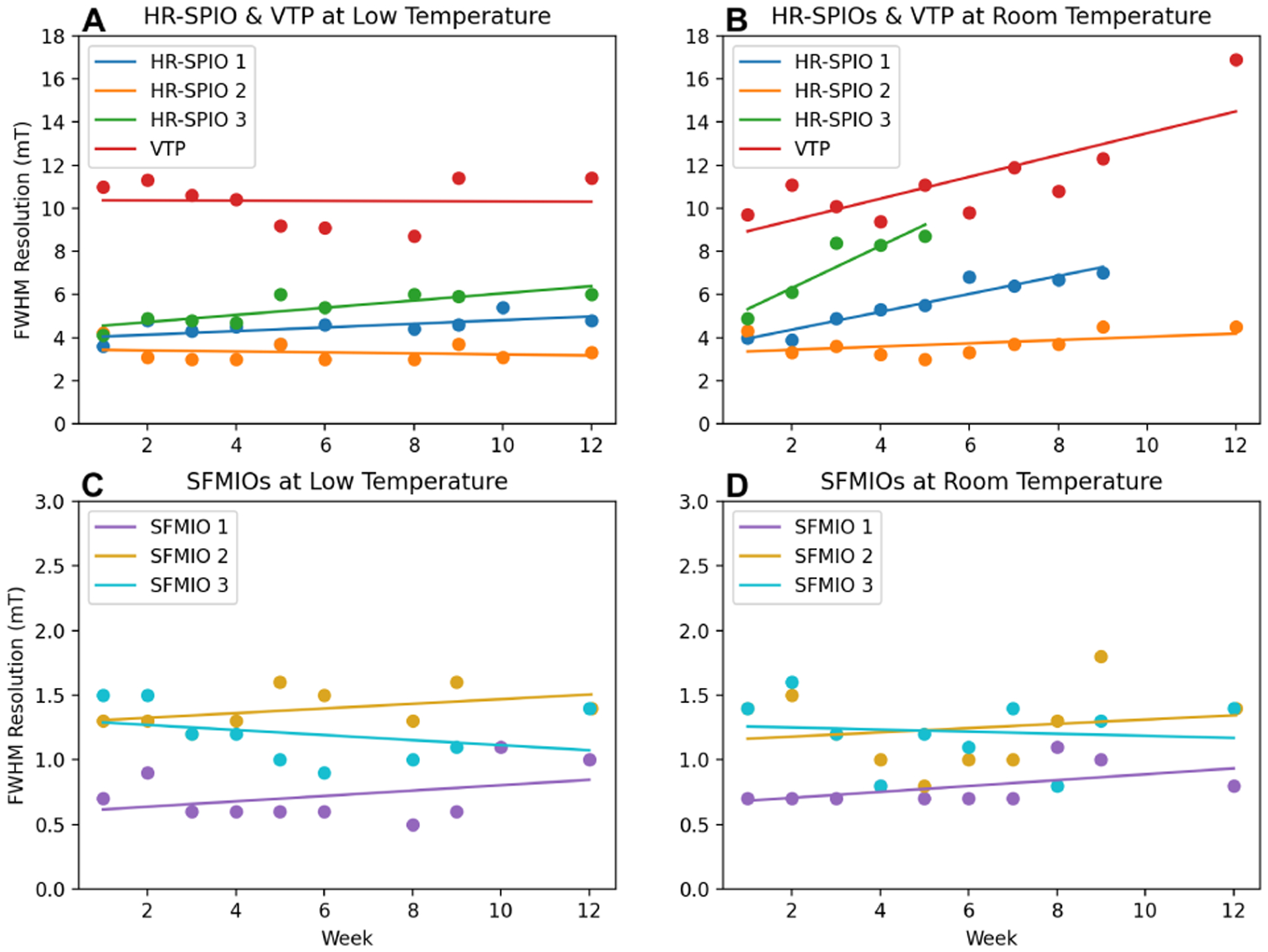
Comparison of HR-SPIOs and SFMIO resolution (rows) at two temperatures (columns) over time. The circular data point represents the lowest FWHM recorded each week (transmit amplitude is not held constant). A least-squares linear regression line is provided for each particle. FWHM values of VTP are reported using the standard transmit amplitude of 20 mT. The lowest FWHM resolution recorded using scan amplitudes between 2–40 mT are reported for all other particles.

**Figure 4:**
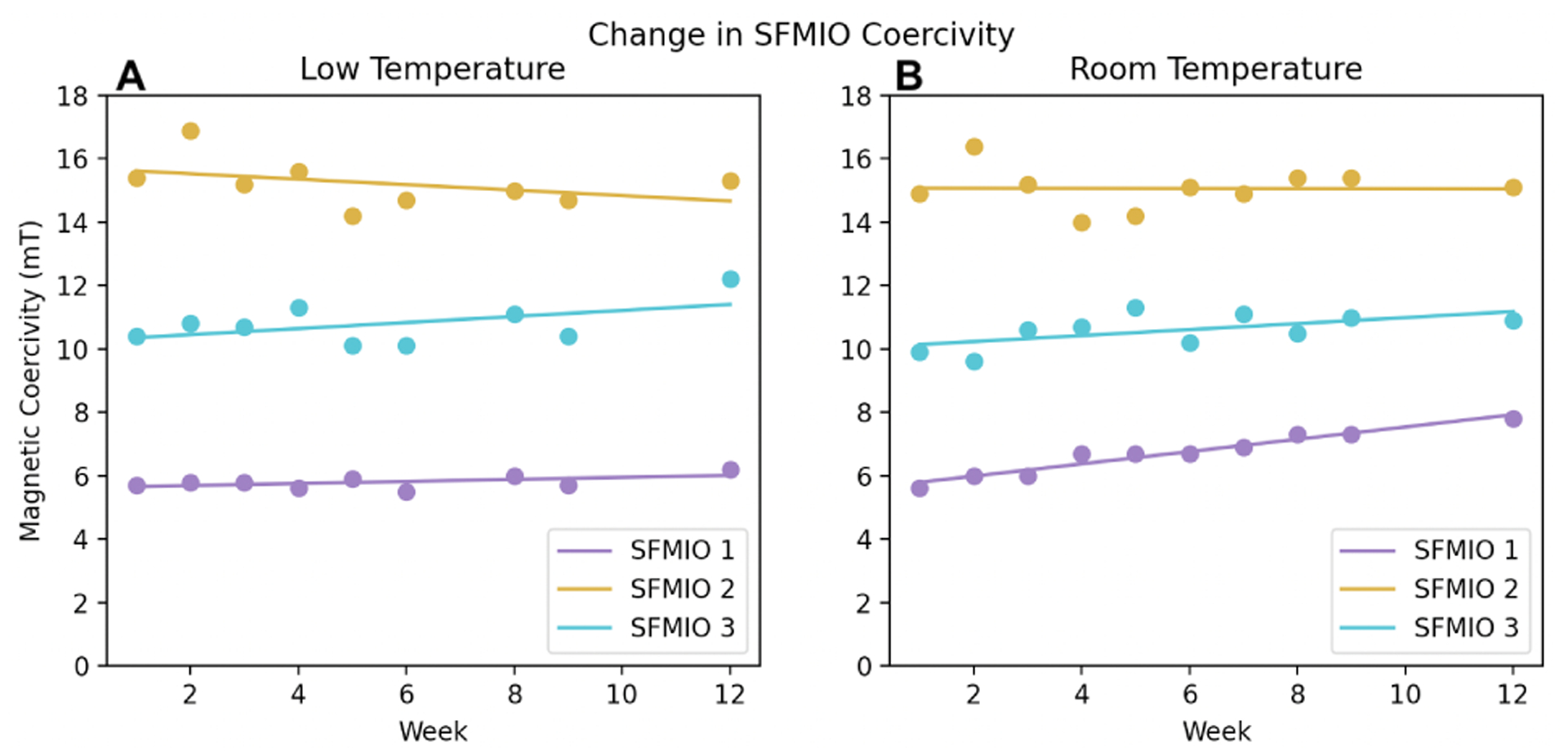
Coercivity of SFMIO particles stored at low and room temperatures. A least-squares linear regression line is provided for each particle. No appreciable difference in coercivity was observed between the two temperature groups.

HR-SPIOs and SFMIOs exhibit different responses to storage temperature. Figures 3A and 3B compare HR-SPIO and VTP resolutions when stored at low temperatures and at room temperature. Particles stored at room temperature show increases in FWHM over time, a worsening of the resolution, while those stored at the lower temperature show minimal resolution degeneration. On the other hand, Figures 3C and 3D reveal that temperature appears to have a negligible impact on SFMIO resolution. All SFMIO resolutions remain below all HR-SPIOs resolutions, even after 12 weeks at room temperature. Coercivity in SFMIOs showed minor differences when stored at different temperatures (Figure 4).

## V. Discussion

The results show that HR-SPIOs are much more prone to degrading in resolution when stored at higher temperatures. While this is a small sample, a linear model fits the HR-SPIO resolution degradation process in the first twelve weeks. Meanwhile, SFMIOs showed no appreciable difference in resolution between storage at low temperatures and room temperature. Similarly, SFMIO coercivity does not seem to be significantly affected by storage temperature. The longevity of SFMIO resolution and coercivity allow for cheap and accessible tracer storage infrastructure compared to nuclear medicine studies such as FDG-PET.

The different responses to temperature over a long duration invite further investigation of how long HR-SPIOs and SMFIOs may be able to maintain their resolution at body temperature. Future clinical applications of MPI such as cancer tracking may benefit from tracers that can be re-imaged weeks after the initial injection with the same resolution, thus minimizing the number of injections. Furthermore, additional environmental factors such as oxidation should be considered for future MPI applications and elucidating the role of oxidation in particle longevity.

## VI. Conclusion

By tracking the resolution and coercivity of HR-SPIOs and SFMIOs, we uncovered that SFMIOs appear to be less sensitive to temperature. These results inform researchers of the appropriate storage conditions for their nanoparticle tracers. Furthermore, the persistence of SFMIO resolution and coercivity could make MPI a more accessible technology in the future by not requiring refrigeration in transit or storage for long durations. Future work in this area can employ a similar approach to monitor resolution decay at physiological temperatures and in different oxidative environments.
